# Endoscopic Features of Gastric Epithelial Neoplasm of Fundic Gland Mucosa Lineage

**DOI:** 10.3390/diagnostics12112666

**Published:** 2022-11-02

**Authors:** Kohei Matsumoto, Hiroya Ueyama, Takashi Yao, Tomoyo Iwano, Momoko Yamamoto, Hisanori Utsunomiya, Ryota Uchida, Daiki Abe, Shotaro Oki, Nobuyuki Suzuki, Atsushi Ikeda, Noboru Yatagai, Yoichi Akazawa, Tsutomu Takeda, Kumiko Ueda, Daisuke Asaoka, Mariko Hojo, Akihito Nagahara

**Affiliations:** 1 Department of Gastroenterology, Juntendo University School of Medicine, Tokyo 113-8421, Japan; 2 Department of Human Pathology, Juntendo University Graduate School of Medicine, Tokyo 113-8421, Japan

**Keywords:** gastric adenocarcinoma of fundic gland type, gastric adenocarcinoma of fundic gland mucosa type, gastric epithelial neoplasm of fundic gland mucosa lineage, oxyntic gland adenoma, narrow-band imaging

## Abstract

The endoscopic features of gastric epithelial neoplasms of fundic gland mucosa lineage (GEN-FGML) have not been well investigated. We aimed to clarify the endoscopic features of GEN-FGML and differences between gastric adenocarcinoma of the fundic gland type (GA-FG) and fundic gland mucosa type (GA-FGM). A total of 62 GEN-FGML lesions, including 52 GA-FG and 10 GA-FGM, were retrospectively analyzed using endoscopic and clinicopathological findings to provide information of diagnostic value using white light imaging (WLI) and magnifying endoscopy with narrow-band imaging (M-NBI). GA-FG frequently presented with a whitish, submucosal tumor (SMT) shape with dilated vessels with branching architecture and background mucosa without atrophic change in WLI, an indistinct demarcation line (DL), dilatation of the crypt opening and intervening part (IP), and microvessels without distinct irregularity in M-NBI. GA-FGM frequently presented as a reddish, elevated lesion in WLI, with a distinct DL, dilatation of the IP, and an irregular microvascular pattern in M-NBI. As for an M-NBI diagnosis, five GA-FGM lesions met the diagnostic criteria for cancer, whereas none of the GA-FG lesions met the same criteria. We highlight the endoscopic features of GEN-FGML, and the differentiation between GA-FG and GA-FGM might be possible by combination of lesion color and morphology in WLI and M-NBI diagnoses.

## 1. Introduction

In recent years, many tumors have been reported that have a tendency to differentiate into the fundic gland cells, foveolar epithelial cells, or mucus neck cells, such as oxyntic gland adenoma (OGA), gastric adenocarcinoma of the fundic gland type (GA-FG), and gastric adenocarcinoma of the fundic gland mucosa type (GA-FGM); these are collectively referred to as gastric epithelial neoplasms of a fundic gland mucosa lineage (GEN-FGML) [1,2]. OGA and GA-FG have attracted recent attention, as they were listed as rare gastric neoplasias in the 5th edition of the World Health Organization (WHO) Classification of Tumours, published in 2019 [3]. Compared with OGA and GA-FG, GA-FGM has a higher positive vascular invasion rate and is considered to be a highly malignant tumor [1].

We have reported the four most common endoscopic features of GEN-FGML with white light imaging (WLI) as follows: (1) submucosal tumor (SMT) shape; (2) whitish color; (3) dilated vessels with branching architecture; and (4) background mucosa without atrophic change [4]. In comparison, the four most common endoscopic features of GEN-FGML with magnifying endoscopy with narrow-band imaging (M-NBI) are as follows: (1) indistinct demarcation line (DL) between the lesion and surrounding mucosa; (2) dilatation of the crypt opening (CO); (3) dilatation of the intervening part (IP) between the crypts; and (4) microvessels without distinct irregularity [5]. However, these endoscopic features were analyzed using few numbers of cases that were, additionally, not histopathologically classified into separate GA-FG and GA-FGM categories. It is clinically important to detect GA-FGM, which is considered to have a relatively high malignant potential, and to endoscopically differentiate between GA-FGM and GA-FG. However, endoscopic features, including M-NBI, have not been well investigated, because of few case reports and review articles on the endoscopic features of GEN-FGML [6,7,8,9,10]. In addition, endoscopic diagnosis of GEN-FGML, including differentiation between GA-FG and GA-FGM, is considered very difficult in clinical practice. In the present study, we aimed to clarify the endoscopic features and differences between GA-FG and GA-FGM with WLI and M-NBI and to evaluate the usefulness of endoscopic features that we have reported on previously.

## 2. Materials and Methods

### 2.1. Study Design

A total of 62 GEN-FGML lesions were collected from endoscopic and surgical resections performed between July 2008 and December 2019 at our hospital. We retrospectively analyzed endoscopic and clinicopathological findings of GEN-FGML using WLI and M-NBI and reexamined endoscopic features highlighted in our previous reports in a differential diagnosis between GA-FG and GA-FGM. The 60 lesions analyzed in our previous reports were also included in this study [1]. However, we report the results of this study as novel findings, since our previous reports did not include an analysis of M-NBI in GA-FGM and an analysis of endoscopic differentiation between GA-FG and GA-FGM.

### 2.2. Histopathological Classification of GEN-FGML

All lesions were classified immunohistochemically using a combination of antibodies against MUC5AC (a marker for gastric foveolar epithelial cells), MUC6 (a marker for gastric mucous neck cells), pepsinogen-I, and H+/K+-ATPase. According to its immunohistochemical classification and several previous reports [1,2,11,12], GEN-FGML could be classified into two types, as follows: GA-FG (MUC5AC−, MUC6+ or −, pepsinogen-I, and/or H+K+-ATPase+) and GA-FGM (MUC5AC+, MUC6+ or −, pepsinogen-I, and/or H+K+-ATPase+). We included OGA in GA-FG as an intramucosal lesion of GA-FG in this study, since they share features in terms of endoscopic, histological, immunohistochemical, and molecular biological aspects, and only differ with regard to the depth of invasion. All histology and immunohistochemical staining results were evaluated by a single pathologist (T. Yao) who is specialized in the gastrointestinal tract and one of the authors of the OGA section in the WHO Classification of Tumours, 2019.

### 2.3. Endoscopic and Clinicopathological Assessment

**Figure 1:**
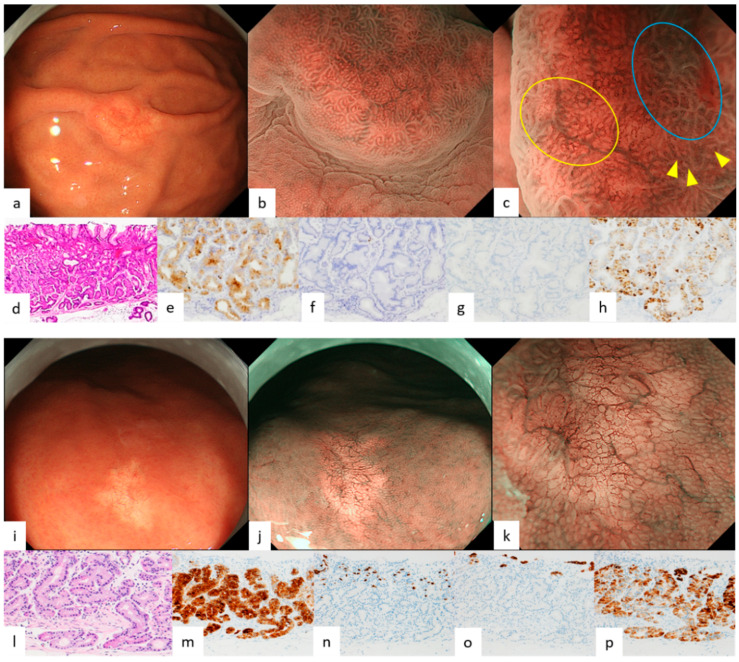
Endoscopic and pathological findings of GA-FG (whitish elevated type, whitish flat or depressed type). (**a**) WLI. An elevated lesion, 15 mm in size, was found in the greater curvature of the upper body of the stomach. Whitish color, SMT shape, and dilated vessels with branching architecture were observed, with no atrophic changes in the background mucosa; (**b**,**c**) M-NBI. Dilatation of CO (yellow arrowhead), dilatation of IP (blue circle), and microvessels without distinct irregularity were observed, but a distinct DL was not observed. These findings are all congruent with our previously reported endoscopic features. Microvessels without distinct irregularity flowed into the collecting venules (yellow circle). A regular MV pattern plus regular MS pattern without a DL was observed by the MESDA-G; the lesion was diagnosed as non-cancerous; (**d**) HE stain. The surface layer was covered by non-neoplastic mucosa, and tumor cells similar to chief cells proliferated primarily in the deep mucosa, showing an irregular branching structure and adhesion. The nuclei of tumor cells were slightly enlarged compared with those of the surrounding non-neoplastic fundic gland tissue; (**e**–**h**) Immunostain; (**e**) pepsinogen-I (chief cell). Diffusely positive; (**f**) H+/K+-ATPase (parietal cell). Partially positive; (**g**) MUC5AC (gastric foveolar epithelial cell). Negative; (**h**) MUC6 (gastric mucous neck cell). Diffusely positive; Final pathological diagnosis: U, type 0-IIa, 23 mm, gastric adenocarcinoma of fundic gland type, pT1b/SM1 (400 μm), UL0, Ly0, V0, HM0, VM0. (**i**) WLI. A whitish flat or depressed lesion 8 mm in size was found on the anterior wall of the greater curvature of the gastric body. Atrophic changes were not noted in the background mucosa, and dilated vessels with branching architecture were found in the surface layer; (**j**,**k**) M-NBI. No distinct DL was noted, and microvessels without distinct irregularity were also observed. A regular MV pattern plus absent MS pattern without a DL were observed by MESDA-G; the lesion was diagnosed as non-cancerous; (**l**) HE staining image. Tumor cells similar to chief cells proliferated primarily in the deep mucosa and partially invaded the submucosal layer up to 50-μm deep; (**m**–**p**) Immunostain; (**m**) pepsinogen-I (chief cell). Diffusely positive; (**n**) H+/K+-ATPase (parietal cell). Partially positive; (**o**) MUC5AC (gastric foveolar epithelial cell). Negative; (**p**) MUC6 (gastric mucous neck cell). Diffusely positive; Final pathological diagnosis: M, type 0-IIb, 7 mm, gastric adenocarcinoma of fundic gland type, pT1b/SM1 (50 µm), UL0, Ly0, V0, HM0, VM0. ((**a**–**c**) cited from [12] Ueyama et al. Gastric adenocarcinoma of fundic gland type. Stomach and Intestine (Tokyo) 2018; 53: 753–767).

Two expert endoscopists (H.U. and K.M.) reviewed endoscopic images of all GEN-FGML lesions. The endoscopic findings of 62 GEN-FGML lesions were investigated retrospectively, according to macroscopic type, color, four endoscopic features with WLI (SMT shape, whitish color, dilated vessels with branching architecture, background mucosa without atrophic change; Figure 1a), four endoscopic features with M-NBI (indistinct DL, dilatation of CO, dilatation of IP, microvessels without distinct irregularity; Figure 1c), and M-NBI diagnosis using the Magnifying Endoscopy Simple Diagnostic Algorithm for early Gastric cancer (MESDA-G) [13]. In addition, we compared GA-FG and GA-FGM via the aforementioned findings of WLI and M-NBI.

The clinicopathological findings included sex, age, treatment, tumor location, tumor size, depth of invasion, median depth of submucosal invasion, presence of vascular invasion, lymph node metastasis, and the status of *Helicobacter pylori* infection. Criteria for the diagnosis of a *H. pylori*-naïve status are described in Supplementary Figure S1.

### 2.4. Statistical Analyses

Continuous variables were compared using Student’s t-test and Mann–Whitney U test. A categorical analysis of variables was performed using a χ2 or Fisher’s exact test when the expected values were less than 5. *p*-values of <0.05 were considered to indicate statistical significance. The statistical program EZR (Easy R; Saitama Medical Center, Jichi Medical University, Saitama, Japan), which is a graphical user interface for R (The R Foundation for Statistical Computing, Vienna, Austria), was used for data analysis.

## 3. Results

Based on the histopathological classification of GEN-FGML, 62 such lesions were subclassified into 52 GA-FG and 10 GA-FGM lesions.

### 3.1. Endoscopic Features of GEN-FGML

**Figure 2:**
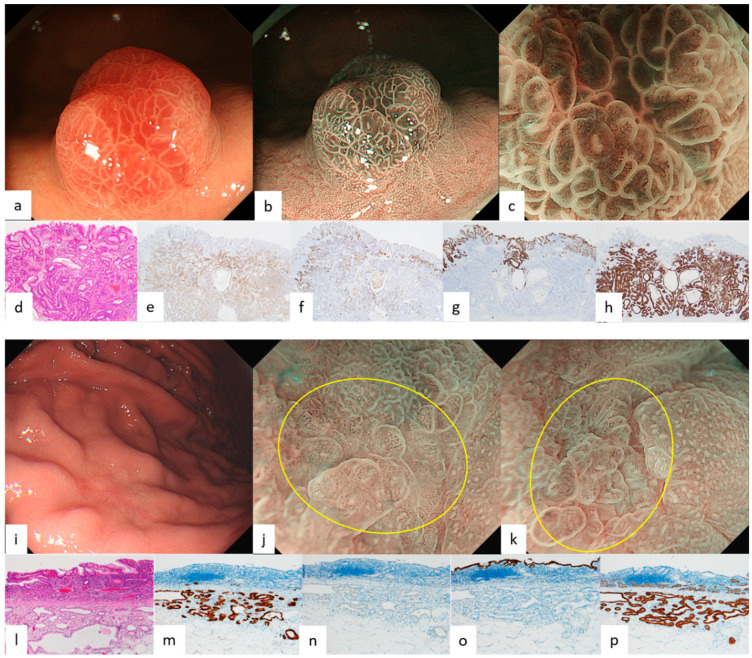
Endoscopic and pathological findings of GA-FG (reddish elevated type, reddish flat or depressed type). (**a**) WLI. An intensely reddish (partially normal-colored) elevated lesion, 12 mm in size, was found in the greater curvature of the gastric fundus. Atrophic changes in the background mucosa, SMT shape, and dilated vessels with branching architecture were not noted; (**b**,**c**) M-NBI. Microvessels without distinct irregularity were found in the dilated IP bordered by arcuate MCE. A regular MV pattern plus regular MS pattern without a DL were observed by the MESDA-G; the lesion was diagnosed as non-cancerous; (**d**) HE stain. Tumor cells similar to chief cells proliferated primarily in the deep mucosa, with the surface layer being covered by non-neoplastic mucosa; (**e**–**h**) Immunostain; (**e**) pepsinogen-I (chief cell). Diffusely positive; (**f**) H+/K+-ATPase (parietal cell). Partially positive; (**g**) MUC5AC (gastric foveolar epithelial cell). Negative; (**h**) MUC6 (gastric mucous neck cell). Diffusely positive; Final pathological diagnosis: U, type 0-I, 12 mm, gastric adenocarcinoma of fundic gland type, pT1b/SM1 (200 µm), UL0, Ly0, V0, HM0, VM0. (**i**) WLI. A slightly reddish (partially normal-colored) depressed lesion 18 mm in size was found on the posterior wall of the greater curvature of the upper gastric body. Atrophic changes in the background mucosa, distinct borders, and dilated vessels with branching architecture in the surface layer were not noted; (**j**,**k**) M-NBI. There was no distinct DL, and an irregular MS pattern and dilatation of CO and IP were observed. As for an MV pattern, some microvessels showed irregularity, while others did not show any distinct irregularity. An irregular MV pattern plus irregular MS pattern without a DL were (yellow circle) observed by the MESDA-G; the lesion was diagnosed as non-cancerous. (**l**) HE stain (border of lesion). Tumor cells similar to mucous neck cells or chief cells proliferated primarily in the deep mucosa. The tumor extended laterally and also invaded the submucosal layer. The surface layer was covered by non-neoplastic mucosa with inflammatory cell infiltration and vascular proliferation, suggesting that the difference in height between the lesion and the background mucosa was generated by replacement of the normal fundic gland by the tumor. The tumor cells invaded up to 400 μm; (**m**–**p**) Immunostain; (**m**) pepsinogen-I (chief cell). Diffusely positive; (**n**) H+/K+-ATPase (parietal cell). Negative; (**o**) MUC5AC (gastric foveolar epithelial cell). Negative; (**p**) MUC6 (gastric mucous neck cell). Diffusely positive; Final pathological diagnosis: U, type 0-IIc, 19 mm, gastric adenocarcinoma of fundic-gland type, pT1b/SM1 (400 µm), UL0, Ly0, V0, HM1, VM0. ((**a**,**c**) cited from [5] Ueyama et al. Establishment of endoscopic diagnosis for gastric adenocarcinoma of fundic gland type (chief cell predominant type) using magnifying endoscopy with narrow-band imaging. Stomach and Intestine (Tokyo) 2015; 50: 1533–1547; (**b**,**i**–**k**): cited from [11] Ueyama et al. Endoscopic features of gastric adenocarcinoma of fundic-gland type. Stomach and Intestine (Tokyo) 2020; 55: 1006–1021).

**Figure 3:**
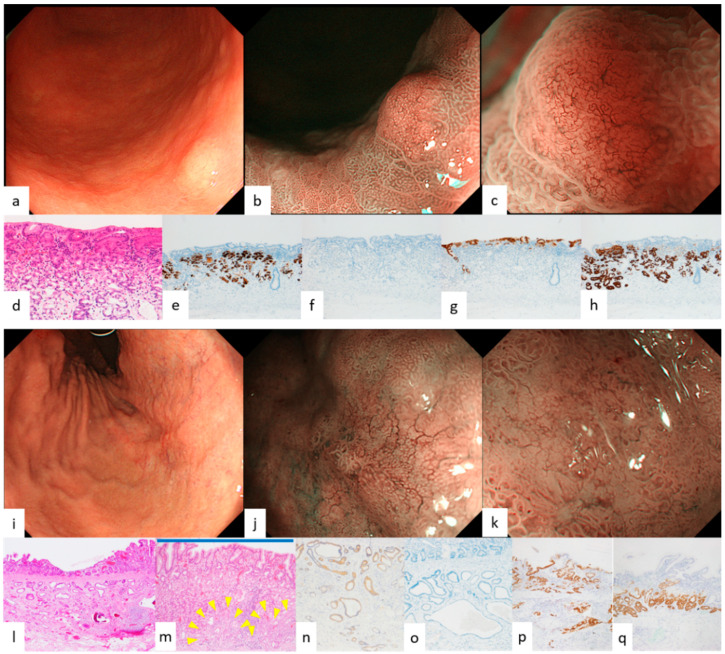
Endoscopic and pathological findings of GA-FGM (whitish elevated type, whitish flat or depressed type). (**a**) WLI. An SMT-like whitish elevated lesion, 5 mm in size, was found on the posterior wall of the greater curvature of the gastric body. Atrophic changes were observed in the background mucosa and no dilated vessels with branching architecture were found; (**b**,**c**) M-NBI. A distinct DL was observed, and microvessels with tortuosity and difference in caliber, if not highly atypical, formed the network. An irregular MV pattern plus absent MS pattern with a DL were observed by the MESDA-G; the lesion was diagnosed as cancerous. (**d**) HE stain. A differentiated adenocarcinoma differentiating into a foveolar epithelium-like structure was exposed on the surface, and tumor cells similar to chief cells proliferated primarily in the middle and deep layers of the mucosa; (**e**–**h**) Immunostain.; (**e**) pepsinogen-I (chief cell). Positive in the middle and deep layers; (**f**) H+/K+-ATPase (parietal cell). Negative; (**g**) MUC5AC (gastric foveolar epithelial cell). Positive in the surface layer; (**h**) MUC6 (gastric mucous neck cell). Positive in the middle and deep layers; Final pathological diagnosis: M, type 0-I, 5 mm, gastric adenocarcinoma of fundic gland mucosa type, pT1a/M, UL0, Ly0, V0, HM0, VM0. (**i**) A whitish depressed lesion, 25 mm in size, with indistinct borders was found in the lesser curvature of the middle body of the stomach. Atrophic changes were observed in the background mucosa, and dilated vessels with branching architecture were observed on the surface; (**j**) M-NBI. The DL was indistinct, and an oval MCE and dilatation of IP were observed; (**k**) M-NBI. An irregular MV plus absent MS pattern without a DL were observed by the MESDA-G; the lesion was diagnosed as non-cancerous; (**l**) HE stain. The tumor extended laterally, primarily in the deep mucosa, and invaded the submucosa over a wide area, up to 1200 μm deep. In the surface layer, a well-differentiated adenocarcinoma existed with low-grade atypia that differentiated into a foveolar epithelium-like structure continuous to the component of fundic gland-type adenocarcinoma; (**m**) HE stain. In some portions of the tumor margin, the surface of the foveolar epithelial-type carcinoma with low-grade atypia (yellow arrowhead) was covered by non-neoplastic mucosa (blue bar); (**n**–**q**) Immunostain; (**n**) pepsinogen-I (chief cell). Diffusely positive; (**o**) H+/K+-ATPase (parietal cell). Negative; (**p**) MUC5AC (gastric foveolar epithelial cell). Positive in the superficial and middle layers; (**q**) MUC6 (gastric mucous neck cell). Positive in the middle layer of the mucosa; Final pathological diagnosis: U, type 0-IIc, 24 mm, gastric adenocarcinoma of fundic gland mucosa type, pT1b/SM2 (1200 µm), UL0, Ly1, V0, HM1, VM0. ((**k**) cited from [5] Ueyama et al. Establishment of endoscopic diagnosis for gastric adenocarcinoma of fundic gland type (chief cell predominant type) using magnifying endoscopy with narrow-band imaging. Stomach and Intestine (Tokyo) 2015; 50: 1533–1547).

**Figure 4:**
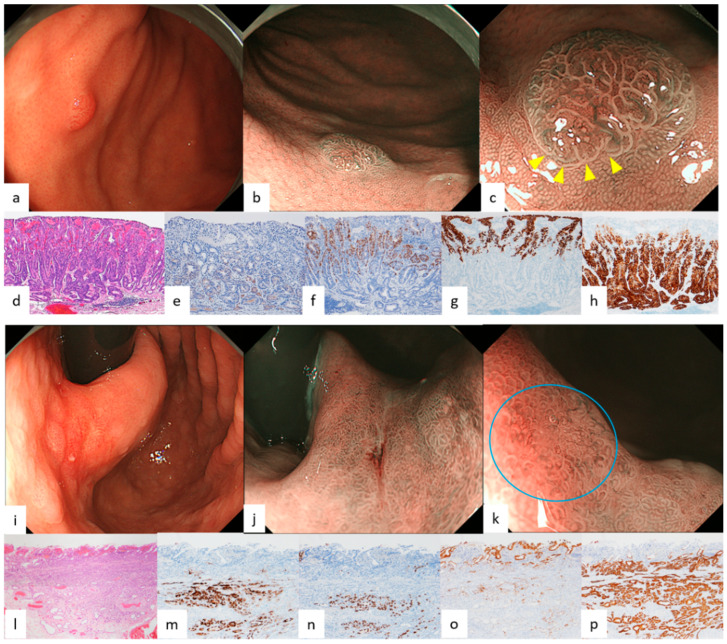
Endoscopic and pathological findings of GA-FGM (reddish elevated type, reddish flat or depressed type). (**a**) WLI. A reddish elevated lesion, 5 mm in size, was found in the posterior surface of the gastric fornix. No atrophic changes were observed in the background mucosa, and borders were distinct. Dilated vessels with branching architecture were found in the surface layer; (**b**,**c**) (M-)NBI. The pit-like structure of the perilesional fundic gland changed into a groove-like structure, making it possible to observe a distinct DL (yellow arrowhead). Irregular microvessels were found in the dilated IP. An irregular MV pattern plus regular MS pattern with a DL were observed by the MESDA-G; the lesion was diagnosed as cancerous; (**d**) HE stain (overall). Differentiated adenocarcinoma differentiating into a foveolar epithelium-like structure was exposed on the surface, and tumor cells similar to chief cells proliferated primarily in the deep layer of the mucosa. (**e**–**h**): Immunostain; (**e**) pepsinogen-I (chief cell). Positive in the middle and deep layers; (**f**) H+/K+-ATPase (parietal cell). Positive in the middle layer; (**g**) MUC5AC (gastric foveolar epithelial cell). Positive in the surface layer; (**h**) MUC6 (gastric mucous neck cell). Positive in the middle and deep layers; Final pathological diagnosis: U, 4 mm, gastric adenocarcinoma of fundic gland mucosa type, T1b/SM1 (300 µm), UL0, Ly0, V0, pHM0, pVM0. (**i**) WLI. A reddish depressed lesion, 20 mm in size, with an indistinct border was seen on the posterior wall of the lesser curvature of the cardiac region. Atrophic changes were present in the background mucosa, as well as dilated vessels with branching architecture on the surface of the lesion; (**j**) M-NBI. There was no distinct DL, but dilatation of CO and IP was observed; (**k**) M-NBI. Irregular microvessels of a non-uniform shape were observed (blue circle). An irregular MV pattern plus regular MS pattern without a DL were observed by the MESDA-G; the lesion was diagnosed as non-cancerous; (**l**) HE stain. Differentiated adenocarcinoma differentiating into a foveolar epithelium-like structure was observed in the surface layer, and a component of gastric adenocarcinoma of fundic gland type in the deep layer. The tumor invaded the submucosal layer up to 1000 μm deep; (**m**–**p**) Immunostain; (**m**) pepsinogen-I (chief cell). Positive in the middle and deep layers; (**n**) H+/K+-ATPase (parietal cell). Positive in the middle and deep layers; (**o**) MUC5AC (gastric foveolar epithelial cell). Positive in the surface and deep layers; (**p**) MUC6 (gastric mucous neck cell). Positive in the middle and deep layers; Final pathological diagnosis: U, 25 mm, gastric adenocarcinoma of fundic gland mucosa type, T1b/SM2 (1000 µm), UL1, Ly1, V1, pHM0, pVM1. ((**a**,**c**) cited from [12] Ueyama et al. Gastric adenocarcinoma of fundic gland type. Stomach and Intestine (Tokyo) 2018; 53: 753–767.).

Endoscopic features of GEN-FGML are shown in Table 1. In all GA-FG lesions, the frequency of endoscopic features we reported previously was more than 50% for each feature. Endoscopic images of GA-FG according to each color and morphology are shown in Figure 1 and Figure 2. Lesions of GA-FG were divided into four groups in descending order of frequency as follows: whitish and elevated type (44%) > whitish and flat or depressed type (35%) > reddish and elevated type (17%) > reddish and flat or depressed type (4%). Particularly with regard to the typical whitish and elevated type (Figure 1a–c), M-NBI showed no distinct DL, but dilatation of CO was observed at the tumor margin, and many lesions were accompanied by dilatation of the IP toward the center, as well as having microvessels without distinct irregularity in the IP. In all GA-FGM lesions, the frequency of endoscopic features we reported previously was less than 50%, except for dilatation of IP (89%, 8/9). Endoscopic images of GA-FGM according to each color and morphology are shown in Figure 3 and Figure 4. Lesions of GA-FGM were divided into four groups in descending order of frequency as follows: reddish and elevated type (50%) > reddish and flat or depressed type (30%) > whitish and elevated type (10%) = whitish and flat or depressed type (10%). Particularly for the typical reddish and elevated type (Figure 4a–c), in M-NBI, these lesions showed a distinct DL, and many were accompanied by dilatation of the IP, as well as irregular microvessels within the IP.

In a comparison of GA-FG and GA-FGM in the WLI findings, the frequencies of whitish elevated lesions (44%: 23/52 vs. 10%: 1/10, *p* < 0.05) and background mucosa without atrophic change (87%: 46/52 vs. 50%: 5/10, *p* < 0.05) were significantly higher for GA-FG. In comparison, the frequency of reddish elevated (17%: 9/52 vs. 50%: 5/10, *p* < 0.05) and reddish flat/depressed (4%: 2/52 vs. 30%: 3/10, *p* < 0.01) lesions were significantly higher for GA-FGM. As for M-NBI findings, the frequency of microvessels without distinct irregularity (84%: 42/50 vs. 22%: 2/9, *p* < 0.01) and showing an indistinct DL (98%: 49/50 vs. 33%: 3/9, *p* < 0.01) was significantly higher in GA-FG than GA-FGM lesions. Therefore, as found for an M-NBI diagnosis, five GA-FGM lesions (5/9, 56%) met the diagnostic criteria for cancer according to the MESDA-G, whereas none of the GA-FG lesions met the same criteria. With regard to GA-FGM, lesions that were diagnosed as cancer by M-NBI were diagnosed because they had significantly irregular MV patterns and a distinct DL compared with lesions that were difficult to diagnose as cancer (Table 2).

### 3.2. Clinicopathological Features of GEN-FGML

Clinicopathological features of GEN-FGML are shown in Table 3. With regard to median tumor size (5.5 mm vs. 17 mm, *p* < 0.05), median depth of submucosal invasion (100 µm vs. 450 µm, *p* < 0.05), and rate of lymphatic invasion (0% vs. 30%, *p* < 0.01), these were significantly higher for GA-FGM than GA-FG lesions. There was no difference in *H. pylori* infection status.

### 3.3. Histopathological and Immunohistochemical Features of GEN-FGML

**Figure 5:**
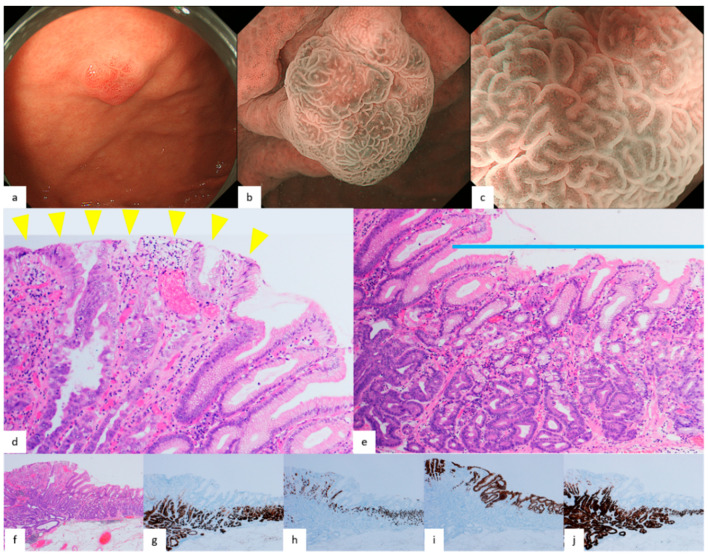
Endoscopic and pathological findings of GA-FGM that with M-NBI diagnosis was non-cancerous. (**a**) WLI. A reddish elevated lesion with SMT-like elevation, 15 mm in size, was noted, and the leading edge of the elevation showed a whitish color; (**b**,**c**) M-NBI. There was no distinct DL, but dilatation of CO and IP was observed. An arcuate MS without distinct irregularity was also noted, and no MV was visible. An absent MV pattern plus regular MS pattern without a DL were observed by the MESDA-G; the lesion was diagnosed as non-cancerous; (**d**) HE stain. Differentiated adenocarcinoma with low-grade atypia differentiating into a foveolar epithelium-like structure was seen in the surface layer (yellow arrowhead); (**e**) HE stain. In some parts, the surface layer was covered by non-neoplastic mucosa (blue bar), and the component of fundic gland-type adenocarcinoma was seen in the middle and deep layers of the mucosa; (**f**) HE stain; (**g**–**j**) Immunostain; (**g**) pepsinogen-I (chief cell). Positive in the middle and deep layers; (**h**) H+/K+-ATPase (parietal cell). Positive in the middle layer; (**i**) MUC5AC (gastric foveolar epithelial cell). Positive in the surface layer; (**j**) MUC6 (gastric mucous neck cell). Positive in the middle and deep layers; ((**a**,**c**) cited from Ueyama et al. gastric adenocarcinoma of fundic gland mucosa type (adenocarcinoma). The Atlas of Upper Gastrointestinal Endoscopic Diagnosis (Tokyo) 2020: 142–143).

Pathological findings of GA-FG and GA-FGM are shown in Figure 1, Figure 2, Figure 3 and Figure 4. For GA-FG, pathological findings showed a proliferation of neoplastic cells similar to fundic gland cells invading from the deep layer to beneath the superficial layer of the mucosa. The superficial layer of the tumor was completely covered with non-neoplastic epithelial cells, with no tumor exposed on the superficial layer. In comparison, in the case of GA-FGM, neoplastic cells differentiating toward a foveolar epithelium were exposed on the superficial layer of the tumor; neoplastic cells similar to fundic gland cells invaded continuously from the portion exposed on the surface to the deep layer of the tumor. Of the lesions that were difficult to diagnose as cancer by M-NBI, 50% (2/4) showed partial superficial coverage by non-neoplastic mucosa, and all (4/4) were differentiated adenocarcinomas with low-grade atypia (Figure 5; Table 2).

The results of immunohistochemical analyses are shown in Table 4. As for the mucin phenotype, according to combinations of the expression of CD10, MUC2, MUC5AC, and MUC6, the frequency of a gastrointestinal phenotype (GA-FG vs. GA-FGM = 0% vs. 20%, *p* < 0.05) was significantly higher for GA-FGM than for GA-FG lesions. As for proliferative activity and p53 overexpression, the mean value of the Ki-67 labeling index (GA-FG vs. GA-FGM = 6% vs. 14%, *p* < 0.01) and the frequency of p53 overexpression (GA-FG vs. GA-FGM = 0% vs. 22%, *p* < 0.05) were significantly higher for GA-FGM than for GA-FG lesions.

## 4. Discussion

This is the first comprehensive report classifying endoscopic features of GEN-FGML into two histological types, namely GA-FG and GA-FGM, and analyzing all possible types based on color combined with morphology. Whitish and elevated lesions were common for GA-FG, as previously reported by others [6,9,14]. Most findings were congruent with endoscopic features in WLI and M-NBI previously reported by us [4,5]. For GA-FGM, however, reddish and elevated lesions predominated; many lesions were diagnosable as cancer by M-NBI and tended toward high clinicopathologic malignancy, with only a limited number of findings being congruent with endoscopic features reported by us [4,5]. We thus found previously highlighted endoscopic features were of value in the diagnosis of GA-FG. Several case reports from others on the endoscopic findings of GA-FGM also indicated the high prevalence of elevated lesions, as in the present study, with many lesions diagnosable as cancer by M-NBI [10]. However, previous reports, including ours, involving a large number of cases taken together indicated lesions that were of a reddish or normal color, and whitish lesions were almost equal in number [1,10,15,16,17,18,19,20,21,22,23]. The reason why GA-FGM lesions tended to take on a reddish color compared with GA-FG lesions could be because such tumors might be exposed on the surface and rich in blood vessels; however, definite reasons remain unidentified. Due to its rarity, GA-FGM has been studied in only a small number of cases, meaning further studies with a large number of cases are warranted.

Of the four features on WLI, SMT shape, whitish color, and dilated vessels with branching architecture are thought to derive from the growth pattern of the tumor under the non-neoplastic epithelium covering the surface layer. All four features are observed in more than 90% of GA-FG lesions, especially in those typically whitish and elevated, and are very useful endoscopic findings for diagnosis. However, it is necessary to understand the characteristics of each type, since these findings tend to be few for GA-FG, which has an atypical color and morphology, such as a reddish color and flat or depressed type. In particular, reddish and of an elevated type lesions (Figure 4a–c) required differentiation from a gastric foveolar-type adenoma (raspberry-shaped gastric cancer in Japan) [24,25]. In contrast, the percentage of GA-FGM lesions showing the above four features was generally low. This was presumably due to GA-FGM showing a different growth pattern from that of GA-FG, with tumor components differentiating into a foveolar epithelium-like structure exposed on the surface. 

We previously described the following four features on M-NBI: indistinct DL, dilatation of IP, dilatation of CO, and microvessels without distinct irregularity. These findings are also thought to stem from the growth pattern of GA-FG, as mentioned above. On magnifying endoscopy according to MESDA-G, GA-FG was diagnosed as non-cancerous in all cases and is considered to be one of the lesions subject to M-NBI diagnostic limitations [26]. With respect to GA-FGM, however, the prevalence of the four features on M-NBI, except for dilatation of IP, was low at about 30%. This was presumably due to the aforementioned tumor components being exposed on the surface, making the irregularity of MV/MS in GA-FGM lesions higher than in GA-FG lesions. Additionally, the pit-like structure of the fundic gland transforms into a groove structure, making DL recognizable (Figure 4b,c). About half of the GA-FGM lesions were diagnosable as cancer by the MESDA-G based on these endoscopic features. However, attention should be paid to lesions showing low-grade atypical changes in tumor components exposed on the surface or with partially covered and/or mixed with a non-neoplastic mucosa, since non-cancerous diagnoses could be made in such lesions due to the low irregularity of MV/MS and the difficulty in recognizing DL (Figure 5b,c). A GA-FGM case with a histological architecture showing complete coverage of the tumor surface by non-neoplastic epithelium has also been reported [1]. While such cases are difficult to differentiate endoscopically from OGA and GA-FG, no such GA-FGM cases were observed in our study.

Regarding the degree of malignancy, GA-FG lesions tended to invade the submucosal layer (73%, 38/52), despite having a small tumor diameter. However, no vascular invasion or lymph node metastasis was noted, suggesting that the tumors were essentially of low grade, as reported previously [1,2]. For GA-FGM tumors, the submucosal invasion rate was comparable to that in GA-FG tumors (80%). However, the tumor diameter was larger, and lymphatic invasion (30%, 3/10), venous invasion (10%, 1/10), and lymph node metastasis (10%, 1/10) were observed. In particular, lymphatic invasion was significantly more common for GA-FGM than GA-FG tumors. GA-FGM may be more malignant than GA-FG, since several reports describe GA-FGM showing vascular invasion and lymph node metastasis [1,16,21,27,28,29]. The therapeutic approach of GA-FG and GA-FGM is currently the same as common-type gastric cancer. However, the additional surgical resection after endoscopic resection might not be indicated for GA-FG cases, since GA-FG exhibited very few vascular invasions and neither recurrence nor metastasis regardless of SM invasion depth. In future, the evaluation of more cases and longer follow-up would be required to confirm the malignant potential of GEN-FGML and draw accurate indications, since there was no difference in the clinical outcomes between GA-FG and GA-FGM cases in previous reports. 

In detecting GEN-FGML, reddish GEN-FGML and typical whitish elevated GA-FG are relatively recognizable. However, small lesions of a whitish flat or depressed type are difficult to identify and often indistinguishable from undifferentiated adenocarcinoma, mucosa-associated lymphoid tissue lymphoma, or focal atrophy. Therefore, particular attention should be paid to cases showing a non-*H. pylori*-infected stomach [30,31]. In cases where the stomach has not been infected with H. pylori, detailed observation should be carried out mainly in the upper and middle thirds of the stomach in order to first identify lesions showing characteristics specific to GA-FG. Even in the absence of such characteristics, GA-FGM should be suspected if the lesion is reddish, has DL on M-NBI, and has an irregular MV pattern or dilatation of IP. It is important to actively undertake a biopsy diagnosis of any suspicious findings; the pathologist should be informed when GEN-FGML is suspected.

The limitations of the present study include its single-center nature, a retrospective design, and the small number of lesions investigated. Further prospective studies conducted in a large number of cases by multiple institutions are necessary for determining accurate endoscopic features of GEN-FGML. Our report [1], which was a multicenter study of GA-FGML, contained the clinicopathological data of 60 lesions in the present study. However, endoscopic features with WLI and M-NBI were not investigated thoroughly. Thus, overlapping lesions with the report did not weaken the scientific novelty of the present study. Detailed endoscopic observation of GEN-FGML is often difficult due to its shape and location in the stomach, especially M-NBI observation, and may require skilled endoscopic techniques such as those of Japanese endoscopists in order to make an accurate diagnosis.

## 5. Conclusions

We have demonstrated endoscopic features and differences between GA-FG and GA-FGM based on color and the construction of the surface and deep portions of tumors. We also showed the usefulness of previously reported endoscopic features with WLI and M-NBI for the accurate and differential diagnosis of GEN-FGML. The results of this study are useful for detection and differentiation of GEN-FGML and clinically beneficial to endoscopists and gastroenterologists. The analysis of GEN-FGML greatly influences the analysis of *H. pylori*-uninfected gastric cancer, which is expected to increase in the future, and we hope that this study will contribute to advanced research on *H. pylori*-uninfected gastric cancer, including GEN-FGML.

## Figures and Tables

**Table 1:** Endoscopic features of GEN-FGML.

	GA-FG (*n* = 52)	GA-FGM (*n* = 10)	*p*-Value
White light image			
Macroscopic type: elevated / flat or depressed	32/20	6/4	0.59
Whitish color/ reddish color	41/11	2/8	<0.01
Four endoscopic features with WLI			
Whitish color	79% (41/52)	20% (2/10)	<0.01
Submucosal tumor shape	56% (29/52)	30% (3/10)	0.17
Dilated vessels with branching architecture	62% (32/52)	50% (5/10)	0.5
Background mucosa without atrophic change	87% (46/52)	50% (5/10)	<0.05
Magnifying endoscopy with NBI			
Four endoscopic features with M-NBI			
Indistinct DL between lesion and surrounding mucosa	98% (49/50)	33% (3/9)	<0.01
Dilatation of crypt opening	64% (32/50)	33% (3/9)	0.13
Dilatation of intervening part between the crypts	94% (47/50)	89% (8/9)	0.49
Microvessels without distinct irregularity	84% (42/50)	22% (2/9)	<0.01
M-NBI diagnosis (MESDA-G) = cancer	0% (0/50)	56% (5/9)	<0.01
Classification according to color and morphology			
Whitish elevated	44% (23/52)	10% (1/10)	<0.05
Whitish flat or depressed	35% (18/52)	10% (1/10)	0.15
Reddish elevated	17% (9/52)	50% (5/10)	<0.05
Reddish flat or depressed	4% (2/52)	30% (3/10)	<0.01

GEN-FGML, gastric epithelial neoplasm of fundic gland mucosa lineage; GA-FG, gastric adenocarcinoma of fundic gland type; GA-FGM, gastric adenocarcinoma of fundic gland mucosa type; WLI, white light image; M-NBI, magnifying endoscopy with narrow-band imaging; DL, demarcation line; MESDA-G, Magnifying Endoscopy Simple Diagnostic Algorithm for early Gastric cancer.

**Table 2:** Endoscopic and pathological findings of GA-FGM according to diagnosis of MESDA-G.

	MESDA-G		*p*-Value
	Cancer (*n* = 5)	Non-Cancer (*n* = 4)	
Endoscopic findings			
Macroscopic type: elevated / flat or depressed	3/2	2/2	0.76
Color: Reddish/ Whitish	4/1	3/1	0.85
Tumor size, mm, median	5	19.5	0.22
Location: Upper/ Middle/ Lower	3/2/0	3/1/0	0.63
Indistinct DL between lesion and surrounding mucosa	0% (0/5)	75% (3/4)	<0.01
Dilatation of crypt opening	20% (1/5)	50% (2/4)	0.49
Dilatation of intervening part between the crypts	80% (4/5)	100% (4/4)	0.34
Microvessels without distinct irregularity	0% (0/5)	50% (2/4)	0.07
Microvascular pattern (regular/irregular/absent)	0/5/0	1/1/2	<0.01
Microsurface pattern (regular/irregular/absent)	2/2/1	3/0/1	0.34
Dilated vessels with branching architecture	40% (2/5)	75% (3/4)	0.29
Background mucosa with atrophic change	60% (3/5)	50% (2/4)	0.76
Pathological findings			
Partially covered with a non-neoplastic mucosa	40%(2/5)	50% (2/4)	0.76
Well-differentiated adenocarcinoma with low-grade atypia	20% (1/5)	100% (4/4)	<0.01

GA-FGM, gastric adenocarcinoma of fundic gland mucosa type; DL, demarcation line; MESDA-G, Magnifying Endoscopy Simple Diagnostic Algorithm for early Gastric cancer.

**Table 3:** Clinicopathological features of GEN-FGML.

	GA-FG (*n* = 52)	GA-FGM (*n* = 10)	*p*-Value
Male/Female	30/22	7/3	0.36
Age, years, average (range)	67.6 (46–87)	65.1 (40–85)	0.46
Treatment: ESD/EMR/OPE	46/5/1	6/3/1	0.47
Location: Upper/Middle/Lower	40/11/1	6/3/1	0.86
Tumor size, mm, median (range)	5.5 (1–43)	17 (4–39)	<0.05
Depth of invasion: M/SM	14/38	2/8	0.94
Median depth of SM invasion, µm (range)	100 (50–1400)	450 (200–1500)	<0.05
Lymphatic invasion	0% (0/52)	30% (3/10)	<0.01
Venous invasion	0% (0/52)	10% (1/10)	0.16
Lymph node metastasis	0% (0/52)	33% (1/3)	0.16
*H.pylori* infection status: naïve/post eradication/positive	34/13/4 (*n* = 51)	5/4/0 (*n* = 9)	0.79

GEN-FGML, gastric epithelial neoplasm of fundic gland mucosa lineage; GA-FG, gastric adenocarcinoma of fundic gland type; GA-FGM, gastric adenocarcinoma of fundic gland mucosa type; ESD, endoscopic submucosal dissection; EMR, endoscopic mucosal resection; OPE, operation; M, mucosa; SM, submucosa.

**Table 4:** Immunohistochemical features of GEN-FGML.

	GA-FG (*n* = 52)	GA-FGM (*n* = 10)	*p*-Value
Cell differentiation			
pepsinogen-I	100% (52/52)	100% (10/10)	1
H+/K+-ATPase (including focal positive)	94% (49/52)	100% (10/10)	1
MUC5AC	0% (0/52)	100% (10/10)	<0.01
MUC6	96% (50/52)	100% (10/10)	1
MUC2	0% (0/52)	0% (0/10)	1
CD10	0% (0/52)	20% (2/10)	<0.05
Mucin phenotype			
Gastric phenotype	98% (51/52)	80% (8/10)	0.22
Gastrointestinal phenotype	0% (0/52)	20% (2/10)	<0.05
Intestinal phenotype	0% (0/52)	0% (0/10)	1
Unclassified phenotype	2% (1/52)	0% (0/10)	1
p53 over expression	0% (0/48)	22% (2/9)	<0.05
Ki-67 MIB1 LI, average (range)	6% (1–40%)	14% (1–30%)	<0.01

GEN-FGML, gastric epithelial neoplasm of fundic gland mucosa lineage; GA-FG, gastric adenocarcinoma of fundic gland type; GA-FGM, gastric adenocarcinoma of fundic gland mucosa type.

## Data Availability

Not applicable.

## Supplementary Materials

The following supporting information can be downloaded at: https://www.mdpi.com/article/10.3390/diagnostics12112666/s1, Figure S1: Criteria for diagnosis of *H. pylori*-naïve status.
