# Supplementary material for: Endoscopic Features of Gastric Epithelial Neoplasm of Fundic Gland Mucosa Lineage
Source: Diagnostics (Basel). 2022 Nov 2;12(11):2666. doi: 10.3390/diagnostics12112666 (PMC9689643; doi:10.3390/diagnostics12112666)
Supplement: Supplementary file 1 [file diagnostics-12-02666-s001.zip › diagnostics-1935295-supplementary.pdf]

Criteria for diagnosis of *H.pylori*-naïve status

Endoscopic findings	No mucosal atrophy (no atrophic change or C-1 according to Kimura-Takemoto classification)
Pathological findings	No atrophic change in resected specimen
Clinical findings	Negativity in one or more tests (e.g. RUT, UBT, serum IgG, stool antigen)
History of eradication of *H.pylori*	None

RUT: rapid urease test, UBT: urea breath test

Figure S1: Criteria for diagnosis of *H.pylori*-naïve status
